# Hypothalamic–pituitary–adrenal axis activity and neurotrophic factors in drug-naive children and adolescents with attention-deficit/hyperactivity disorder

**DOI:** 10.3389/fpsyt.2026.1774449

**Published:** 2026-03-11

**Authors:** Hurşit Ferahkaya, Necati Uzun, Hasibe Ağır, İbrahim Kılınç, Abdullah Akkuş, Fatma Coşkun, Ömer Faruk Akça, Ayhan Bilgiç

**Affiliations:** 1 Department of Child and Adolescent Psychiatry, School of Medicine, Necmettin Erbakan University, Konya, Türkiye; 2 Department of Biochemistry, School of Medicine, Necmettin Erbakan University, Konya, Türkiye; 3 Department of Pediatrics, School of Medicine, Necmettin Erbakan University, Konya, Türkiye; 4 Department of Child and Adolescent Psychiatry, Izmir University of Economics, İzmir, Türkiye

**Keywords:** adrenocorticotropic hormone, attention-deficit/hyperactivity disorder, cortisol, HPA axis, neurotrophins

## Abstract

**Background:**

Attention deficit/hyperactivity disorder (ADHD) is a neurodevelopmental disorder with a complex and not fully understood etiology. Increasing evidence suggests that neurotrophic factors involved in neurodevelopment and synaptic plasticity, as well as hormones of the hypothalamic-pituitary-adrenal (HPA) axis that regulate the stress response, may contribute to the pathophysiology of ADHD.

**Methods:**

This cross-sectional study aimed to compare children diagnosed with ADHD and healthy controls with respect to serum levels of brain-derived neurotrophic factor (BDNF), glial cell line–derived neurotrophic factor (GDNF), vascular endothelial growth factor (VEGF), neurotrophin-3 (NT-3), adrenocorticotropic hormone (ACTH), and cortisol. A total of 80 children aged 6–18 years with a diagnosis of ADHD and 81 healthy controls were included in the study. The severity of ADHD symptoms was assessed using the Conners’ Parent Rating Scale–Short Version (CPRS-SV). Serum levels of biochemical parameters were measured using commercially available electrochemiluminescence immunoassay and enzyme-linked immunosorbent assay kits.

**Results:**

Compared with the healthy control group, the ADHD group exhibited significantly higher serum levels of BDNF, GDNF, VEGF, ACTH, and cortisol, whereas NT-3 levels did not differ between the groups. These group differences remained statistically significant after controlling for potential confounding variables. Correlation analyses revealed no significant associations between neurotrophic factors, hypothalamic–pituitary–adrenal (HPA) axis hormones, and CPRS-SV subscale scores.

**Conclusions:**

The present findings indicate that neurotrophic factors and hormones related to the hypothalamic–pituitary–adrenal (HPA) axis are altered in medication-naïve children and adolescents with ADHD. The absence of a direct correlation between neurotrophic factors and HPA axis hormones suggests that these systems may contribute to the pathophysiology of ADHD through parallel yet partially independent and complex mechanisms. Future longitudinal and multimodal studies are warranted to elucidate the dynamic interactions between stress-related neuroendocrine processes and neurodevelopmental pathways in ADHD.

## 1 Introduction

Attention-deficit/hyperactivity disorder (ADHD) is a neurodevelopmental disorder characterized by inattention, hyperactivity, and impulsivity, which adversely affect an individual’s functioning (1). ADHD has been reported to occur in approximately 3–10% of school-aged children, yet its etiology has not been fully elucidated (2–4). Current evidence suggests that the etiopathogenesis of ADHD involves an interaction between genetic susceptibility, environmental factors, and neurobiological mechanisms (5). Among these neurobiological mechanisms, neurotrophic factors—particularly those associated with neurodevelopment and synaptic plasticity—and hormones of the hypothalamic–pituitary–adrenal (HPA) axis, which are key regulators of the stress response, have attracted increasing research interest in recent years (4, 6–9).

Neurotrophic factors are proteins that regulate processes such as neuronal proliferation, differentiation, synaptogenesis, and cellular survival, and are therefore critical for neurodevelopment (10). Among these factors, brain-derived neurotrophic factor (BDNF) is one of the most extensively studied and occupies a central position in ADHD research due to its fundamental role in synaptic plasticity and learning processes. However, findings regarding peripheral BDNF levels in ADHD are inconsistent. While some studies have reported increased BDNF levels (6, 7, 11), others have found decreased levels (12, 13) or no significant differences between groups (4, 14–16). Beyond peripheral circulating levels of BDNF, meta-analyses have supported a potential role of BDNF polymorphisms in the etiopathogenesis of ADHD (17, 18). Overall, although existing evidence suggests that BDNF may contribute to the pathophysiology of ADHD, the underlying mechanisms remain unclear.

Most studies investigating glial cell line–derived neurotrophic factor (GDNF), which plays a critical role in the development and survival of dopaminergic neurons, have reported increased GDNF levels in ADHD (4, 6, 19, 20); however, studies demonstrating decreased GDNF levels have also been published (13). Moreover, the synergistic interaction between GDNF and BDNF suggests that these neurotrophic factors may jointly contribute to the pathophysiology of ADHD (21). Studies examining the relationship between vascular endothelial growth factor (VEGF), which plays a central role in angiogenesis and cerebrovascular function, and ADHD are relatively limited. While two studies reported no significant differences in VEGF levels between groups (22, 23), one study demonstrated reduced VEGF levels in the ADHD group (20). Evidence regarding neurotrophin-3 (NT-3), which is involved in neuronal survival, differentiation, and synaptogenesis, is also limited in ADHD samples. Of the two available studies, one reported increased NT-3 levels (4), whereas the other found no differences between groups (20). In addition, an adult study demonstrated an association between NT-3 polymorphisms and ADHD (24).

The hypothalamic–pituitary–adrenal (HPA) axis is one of the principal neuroendocrine systems regulating the organism’s stress response. This response is mediated through the release of corticotropin-releasing hormone (CRH) from the hypothalamus, which stimulates the secretion of adrenocorticotropic hormone (ACTH) from the pituitary gland, followed by the release of cortisol from the adrenal cortex (25). Recent studies suggest that HPA axis functioning may be altered in ADHD, and that differences in ACTH and cortisol levels may be associated with stress responsiveness, attentional processes, and behavioral regulation (26, 27). A recent study reported decreased morning cortisol levels in individuals with ADHD (27). Consistently, systematic reviews and meta-analyses have demonstrated reduced cortisol levels in the ADHD group (8, 28). In contrast to these findings, some studies have reported increased cortisol levels in specific subgroups or during long-term assessments (29). Although cortisol levels have been extensively investigated in ADHD, studies assessing ACTH levels are relatively scarce, and these studies have not reported significant differences in ACTH levels (26, 30, 31).

The interaction between neurotrophic factors and HPA axis hormones has emerged as a novel area of interest in ADHD research. An animal study demonstrated a reduction in BDNF mRNA levels following the administration of dexamethasone (32). In a study on pituitary adenomas, findings suggested a potential association between the HPA axis and GDNF and VEGF levels (33). Studies simultaneously examining these two biological systems in psychiatric disorders are extremely limited. Bilgiç et al. reported elevated BDNF and ACTH levels in obsessive–compulsive disorder and identified strong negative correlations between BDNF, nerve growth factor (NGF), and NT-3 levels and ACTH and cortisol levels (34). In ADHD, only a single study has evaluated neurotrophic factors and HPA axis hormones concurrently. That study reported low BDNF levels and reduced bedtime cortisol levels, while cortisol levels at the other three time points did not show significant differences. The relationship between BDNF and cortisol was not examined in that study (35).

Taken together, these findings indicate that studies simultaneously examining both neurotrophic factors and HPA axis hormones in ADHD are extremely limited, highlighting a significant research gap in understanding the potential interactions between these biological systems. In the present study, we aimed to concurrently evaluate the levels of neurotrophic factors (BDNF, GDNF, VEGF, and NT-3) and HPA axis hormones (cortisol and ACTH), as well as the possible associations between these two groups of molecules in ADHD. Based on previous evidence reporting alterations in neurotrophic factor levels and HPA axis functioning in ADHD, we hypothesized that (1) neurotrophin and HPA axis hormone levels would differ between the ADHD group and healthy controls, (2) alterations in these molecules would be associated with the clinical characteristics of ADHD, and (3) significant relationships would be observed between neurotrophic factor levels and cortisol and ACTH levels.

## 2 Materials and methods

### 2.1 Participants

The patient group consisted of children and adolescents aged 6–18 years who were diagnosed with ADHD and consecutively admitted to the Outpatient Clinic of the Department of Child and Adolescent Psychiatry, Necmettin Erbakan University Faculty of Medicine. Exclusion criteria for the patient group were defined under three categories: (1) Psychiatric exclusion criteria: the presence of any psychiatric disorder other than ADHD, including major depressive disorder, anxiety disorders, bipolar disorder, autism spectrum disorder, schizophrenia, obsessive–compulsive disorder, and conduct disorder; (2) Medical exclusion criteria: a history of severe head trauma, organic brain damage, genetic syndromes, or respiratory, metabolic, endocrine, or neurological diseases; (3) Medication-related exclusion criteria: a history of receiving ADHD treatment at any time during the lifetime or the use of psychotropic medications within the past 6 months. The control group included children and adolescents aged 6–18 years who were admitted to the pediatric outpatient clinic for routine follow-up visits (e.g., height–weight monitoring, health screening), had no current or past diagnosis of any psychiatric disorder, including ADHD, had not experienced an acute infection within the past month, and had no history of chronic organic disease.

Ethical approval for the study was obtained from the Ethics Committee of Necmettin Erbakan University (approval no. 2024/5223). After the research procedures were explained, written and verbal informed consent was obtained from the participants and their parents. A total of 109 children and adolescents with ADHD were assessed; 18 declined to participate, and 11 were excluded according to the exclusion criteria. In the control group, 100 participants were evaluated; 9 declined to participate, and 10 were excluded due to the exclusion criteria.

### 2.2 Diagnosis and symptom assessment

ADHD diagnoses were established according to the criteria of the Diagnostic and Statistical Manual of Mental Disorders, Fifth Edition (DSM-5) (36). Psychiatric evaluations of all participants in both groups were conducted by a certified and experienced child and adolescent psychiatrist using the Turkish version of the Schedule for Affective Disorders and Schizophrenia for School-Age Children—Present and Lifetime Version (K-SADS-PL). The severity of symptoms in the ADHD group was assessed using the Revised Conners’ Parent Rating Scale–Short Form (CPRS-SV). Turkish validity and reliability studies for both assessment instruments have been established (37, 38).

### 2.3 Blood samples

All participants were instructed to avoid strenuous physical activity on the day preceding the assessment to prevent potential effects on biochemical parameters. Following an overnight fast of 8 hours, a total of 10 mL of venous blood was drawn from the antecubital vein between 08:30 and 09:30 a.m. The collected blood samples were transferred into biochemical tubes, and serum was separated by centrifugation at 1,000 × g for 10 minutes at 4 °C. The obtained serum samples were stored at −80 °C until analysis. Serum concentrations of BDNF, GDNF, VEGF, and NT-3 were measured using enzyme-linked immunosorbent assay (ELISA) according to the protocols recommended by the manufacturers (SUNLONG: SL0371HU, SL0756HU; YLBIONT: YLA1208HU; SUNRED: 201-12-1306-96T, respectively). Serum cortisol and plasma ACTH levels were measured using an electrochemiluminescence immunoassay (ECLIA) method on a Roche Cobas e801 analyzer (Roche Diagnostics GmbH, Mannheim, Germany), employing the manufacturer’s kits, calibrators, and quality control materials.

### 2.4 Statistical analysis

Statistical analyses were performed using IBM SPSS Statistics version 23.0 (IBM Corp., Armonk, NY, USA). The normality of continuous variables was assessed using the Shapiro–Wilk test. Descriptive statistics are presented as mean ± standard deviation for continuous variables and as frequencies and percentages for categorical variables. Comparisons of categorical variables between the ADHD and control groups were conducted using the chi-square test. Due to distributional characteristics, group differences in continuous variables were analyzed using the Mann–Whitney U test. Serum levels of BDNF, GDNF, VEGF, NT-3, ACTH, and cortisol were initially compared between the ADHD and control groups using the Mann–Whitney U test. To meet the assumptions of multivariate analyses and reduce skewness, biochemical variables were logarithmically transformed prior to further analyses.

To control for the potential confounding effects of age, sex distribution, and BMI percentile, and to reduce the risk of type I error due to multiple comparisons, a multivariate analysis of covariance (MANCOVA) was performed with group (ADHD *vs*. control) as the fixed factor and logarithmically transformed serum levels of BDNF, GDNF, VEGF, NT-3, ACTH, and cortisol as dependent variables. Age, sex, and BMI percentile were included in the model as covariates. Following a significant MANCOVA result, separate univariate analyses of covariance (ANCOVAs) were conducted for each dependent variable, with adjustment for covariates, to determine group differences. Effect sizes were reported as partial eta squared (ηp²). Correlations among variables were assessed using Spearman correlation analysis. All statistical tests were two-tailed, and a p value < 0.05 was considered statistically significant.

## 3 Results

The study population consisted of 80 children with ADHD (56 boys/24 girls) and 81 healthy controls (47 boys/34 girls) (X^2^ = 2.504, p =0.114). The mean age did not differ significantly between the patient (11.15 ± 3.09 years) and control (11.62 ± 3.21 years) groups (z=-0.908, p = 0.364). There was no significant difference in BMI percentiles between the patient (64.9 ± 29.7) and control (60.6 ± 33.7) groups (z = -0.490, p = 0.624). The demographic and clinical characteristics of the children with ADHD and controls are summarized in **Table 1**.

**Table 1:** Demographic and clinical characteristics of children with attention-deficit/hyperactivity disorder and controls.

Variables	ADHD (n = 80)	Controls (n = 81)	z/*x^2^*	p
Age, years	11.15±3.09	11.62±3.21	-0.908^a^	0.364
Sex, male/female	56/24	47/34	2.504^b^	0.114
BMI percentile	64.9±29.7	60.6±33.7	-0.490^a^	0.624
Mother education, years	9.0±3.7	8.9±4.3	-0.378^a^	0.706
Father education, years	10.5±3.7	10.5±4.3	-0.050^a^	0.960
CPRS-SV Oppositional Tendencies	8.8±4.9	–	–	–
CPRS-SV Cognitive Problems/Inattention	10.8±4.6	–	–	–
CPRS-SV Hyperactivity	7.5±4.8	–	–	–
CPRS-SV ADHD Index	20.7±7.5	–	–	–

ADHD, attention-deficit hyperactivity disorder; BMI, body-mass index; CPRS-SV, Conners’ Parent Rating Scale-Short Version (Revised).

a Mann-Whitney U test.

b Chi square test.

**Figure 1:**
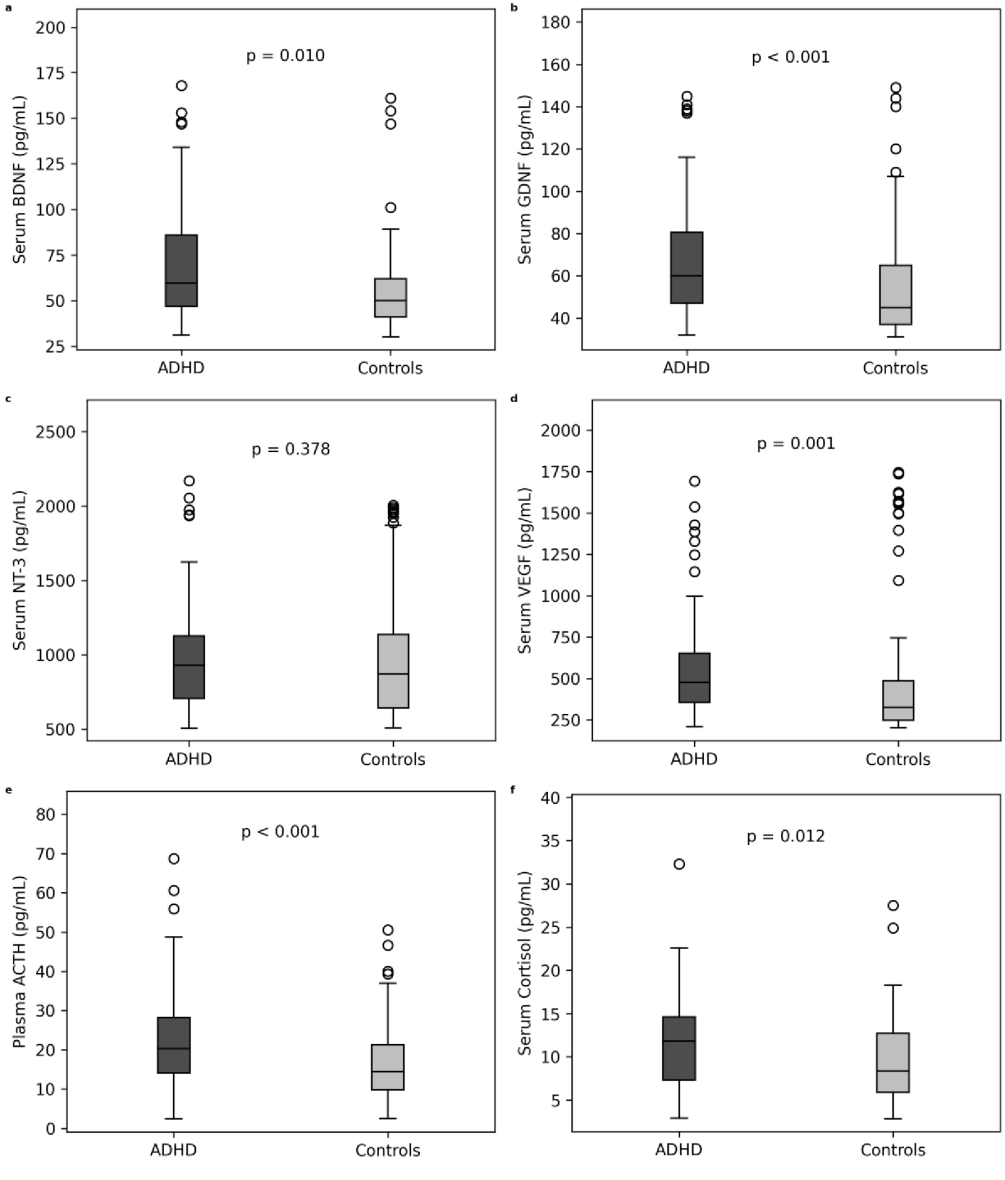
Comparison of serum and plasma biomarker levels between children and adolescents with attention-deficit/hyperactivity disorder (ADHD) and healthy controls. Boxplots represent median values and interquartile ranges, with whiskers indicating minimum and maximum values excluding outliers. **(a)** Serum BDNF, **(b)** Serum GDNF, **(c)** Serum NT-3, **(d)** Serum VEGF, **(e)** Plasma ACTH, and **(f)** Serum cortisol levels (all units: pg/mL). Group comparisons were performed using the Mann-Whitney U test.

Serum BDNF, GDNF, VEGF, ACTH, and cortisol levels were significantly higher in the ADHD group than the control group (z = 2.590, p = 0.010; z = 3.727, p< 0.001; z = 3.183, p = 0.001; z = 3.469, p = 0.001; z = 2.521, p = 0.012, respectively). However, there were no significant differences in serum levels of NT-3 levels in the patient and control groups (z = 0.884, p = 0.377). The mean values of neurotrophins, VEGF, ACTH, and cortisol levels for the patient and control groups are given in **Table 2** and **Figure 1**.

**Table 2:** Serum neurotrophins, VEGF, ACTH, and cortisol levels of children with ADHD and unaffected comparison subjects (Mann-Whitney U Test).

Variables	ADHD (n = 80)	Controls (n = 81)	z	p
BDNF (pg/ml)	69.18±32.6	56.51±24.39	2.590	0.010
GDNF (pg/ml)	67.11±27.57	54.86±26.12	3.727	<0.001
NT-3 (pg/ml)	994.41±385.41	988.90±449.21	0.884	0.377
VEGF (pg/ml)	568.73±330.83	517.81±442.93	3.183	0.001
ACTH (pg/ml)	23.11±12.85	16.93±9.79	3.469	0.001
Cortisol (pg/ml)	11.56±5.35	9.63±4.79	2.521	0.012

BDNF, brain-derived neurotrophic factor; GDNF, glial-derived neurotrophic factor, NT-3, Neurotrophin-3; VEGF, vascular endothelial growth factor; ACTH, adrenocorticotropic hormone.

To avoid the type I error due to a multiple testing effect and to control confounding factors including gender distribution, age, and BMI percentile, MANCOVA test was also conducted. It was determined that there are significant differences between the groups in MANCOVA test. (Pillai’s trace V = 0.371, F = 4.571, p = 0.001, ηp² = 0.154). Separate univariate ANCOVAs were used after adjusted for gender distribution, age, and BMI percentile for comparisons between two groups. Analyses showed significantly higher serum log-BDNF (F = 6.642, p = 0.012, = 0.040), log-GDNF (F = 11.576, p = 0.001, ηp² = 0.069), log-VEGF (F = 4.477, p = 0.036, ηp² = 0.028) log-ACTH (F = 11.449, p = 0.001, ηp² = 0.068), and log-cortisol (F = 5.053, p = 0.026, ηp² = 0.031) levels in the ADHD patient compared with controls. The averages of the logarithmic values of serum neurotrophins, VEGF, ACTH, and cortisol levels are given in **Table 3**.

**Table 3:** Comparison of serum neurotrophins, VEGF, ACTH, and cortisol levels levels of children with attention-deficit/hyperactivity disorder and unaffected comparison subjects according to ANCOVA.

Variables	ADHD (n = 80)	Controls (n = 81)	ANCOVA		
			F	P	ηp²
Log-BDNF(pg/ml)	1.80±0.19	1.72±0.15	6.642	0.012	0.040
Log-GDNF (pg/ml)	1.79±0.17	1.70±0.17	11.576	0.001	0.069
Log-NT-3 (pg/ml)	2.97±0.15	2.96±0.15	0.130	0.719	0.001
Log-VEGF (pg/ml)	2.69±0.22	2.61±0.28	4.477	0.036	0.028
Log-ACTH (pg/ml)	1.30±0.25	1.16±0.25	11.449	0.001	0.068
Log-cortisol (pg/ml)	1.01±0.21	0.93±0.21	5.053	0.026	0.031

BDNF, brain-derived neurotrophic factor; GDNF, glial-derived neurotrophic factor, NT-3, Neurotrophin-3; VEGF, vascular endothelial growth factor; ACTH, adrenocorticotropic hormone.

The correlation between the mean serum neurotrophins, VEGF, ACTH, and cortisol levels and the mean CPRS-SV subscale scores was examined. No association was found for these biochemical levels and psychological test scores.

## 4 Discussion

In this study, peripheral neurotrophic factors and HPA axis hormones were evaluated concurrently in medication-naïve children and adolescents diagnosed with ADHD. The findings demonstrated that serum levels of BDNF, GDNF, VEGF, ACTH, and cortisol were significantly higher in the ADHD group compared with the healthy control group, whereas no significant difference was observed between groups with respect to NT-3 levels. These group differences remained significant after controlling for potential confounding variables, including age, sex, and BMI percentile. No significant associations were found between HPA axis hormones and neurotrophic factors, nor between biochemical parameters and CPRS-SV subscale scores. Overall, our findings point to a potential role of BDNF, GDNF, VEGF, and HPA axis activity in the pathophysiology of ADHD.

In our study, serum BDNF levels were significantly increased in the ADHD group compared with the healthy control group, a finding that is consistent with previous studies reporting elevated peripheral BDNF levels in ADHD (6, 7, 11, 39, 40). In the literature, this increase has been interpreted as a potential compensatory response to functional impairments in dopaminergic and serotonergic pathways reported in ADHD. In addition, we observed elevated ACTH and cortisol levels in the ADHD group. This finding suggests that increased BDNF levels may function as a compensatory mechanism against the potential adverse effects of elevated glucocorticoids, such as cortisol, on neuronal structures. Indeed, Jeanneteau et al. emphasized the complex interaction between BDNF and glucocorticoids at the hypothalamic level, demonstrating that alterations in BDNF signaling may influence HPA axis sensitivity and neuroplasticity (41). In this context, elevated cortisol levels observed in ADHD may be interpreted alongside an increase in BDNF as a compensatory response aimed at counterbalancing the potential neuronal effects associated with chronic stress. Nevertheless, the literature also includes studies reporting decreased or unchanged BDNF levels in ADHD. These conflicting findings may be attributable to methodological factors such as differences in sample age range, sex distribution, and ADHD subtypes. For example, a recent study associated reduced BDNF levels observed in the predominantly inattentive ADHD subtype with decreased neuronal plasticity and cognitive functioning (3). Similarly, another study reported lower BDNF levels in the predominantly inattentive ADHD group and demonstrated that a significant increase in BDNF levels following eight weeks of methylphenidate treatment was observed only in this subtype (42). In contrast, Corominas-Roso et al. reported higher serum BDNF levels in adults with the predominantly inattentive subtype of ADHD compared with other subtypes (43). A recent meta-analysis, however, found no significant differences in BDNF levels in the ADHD group and no significant changes associated with treatment (15). Taken together, these findings indicate that the relationship between BDNF and ADHD is complex and heterogeneous. Although current evidence supports BDNF as an important molecule involved in the pathophysiology of ADHD, it also suggests that its utility as a standalone and reliable biomarker of clinical severity may be limited. Ultimately, it remains unclear whether the elevated BDNF levels observed in ADHD reflect a pathological process or a compensatory mechanism; further subtype-specific and longitudinal studies are required to clarify this relationship.

In our study, consistent with a large proportion of the existing literature, serum GDNF levels were found to be significantly increased in the ADHD group compared with the healthy control group (4, 6, 19, 20). GDNF is an important neurotrophic factor with neuroprotective properties that plays a critical role in supporting the survival and differentiation of dopaminergic neurons and in regulating synaptic plasticity, which is essential for learning and memory processes (19). Considering these biological functions, elevated GDNF levels in ADHD may represent a secondary compensatory mechanism in response to impairments in dopaminergic functioning and synaptic plasticity reported in the disorder. However, not all findings in the literature fully align with this interpretation. For instance, a study reporting decreased GDNF levels in the predominantly inattentive ADHD subtype suggested that this reduction may reflect insufficient neural recovery and repair capacity (13). The same study also emphasized that alterations in GDNF levels observed in ADHD may vary according to clinical subtypes of the disorder. In another study, no significant changes in GDNF levels were detected regardless of ADHD subtype (35). When considered together, these contradictory findings suggest that changes in GDNF levels in ADHD may be associated with the clinical heterogeneity and subtype differences of the disorder rather than reflecting a unidirectional and homogeneous biological pattern. In this context, future studies with larger samples and subtype-specific designs are likely to provide important insights into the role of GDNF in the etiopathogenesis of ADHD.

Although vascular endothelial growth factor (VEGF) is primarily known as a growth factor involved in angiogenesis, it is also considered to exhibit neurotrophic properties due to its effects on neuronal survival, neurogenesis, and synaptic plasticity. In the present study, VEGF levels were found to be significantly increased in the ADHD group compared with the healthy control group (22). To date, only three studies have investigated VEGF levels in ADHD. Two of these studies reported no significant differences in VEGF levels between ADHD and control groups (22, 23), whereas one study reported significantly decreased VEGF levels in the ADHD group (20). The failure to control for the potential effects of body mass index (BMI) in these studies may represent one of the possible reasons for the inconsistencies among findings, as BMI is known to influence neurotrophic factor levels (44). In our study, the persistence of statistically significant elevations in VEGF levels after controlling for BMI percentile allows for a more methodologically robust interpretation of our findings. In addition to ADHD, alterations in VEGF and soluble VEGF receptor levels have been reported in autism spectrum disorder (ASD), another neurodevelopmental condition, suggesting that such changes may influence neurodevelopmental processes (45). VEGF has been reported to play a role in maintaining the integrity of the blood–brain barrier, and alterations in VEGF levels may affect barrier permeability, leading to changes in cerebral blood flow and brain microvascular structure (46). These mechanisms support the importance of VEGF in brain development and provide plausible biological pathways that may explain VEGF alterations observed in neurodevelopmental disorders such as ADHD and ASD. In line with this perspective, a study reported that elevated VEGF levels measured during the neonatal period were associated with an increased risk of developing ADHD in later years (47). Taken together, although VEGF is thought to play a potential role in the pathophysiology of ADHD, further studies with larger samples and longitudinal designs are required to elucidate the precise mechanisms underlying the relationship between VEGF and ADHD.

In our study, serum NT-3 levels in the ADHD group did not show a significant difference compared with those in the healthy control group. This finding is consistent with the results reported by Yurteri et al., who also found no significant differences in NT-3 levels in ADHD (20). In contrast, a study conducted by Bilgiç et al. reported increased NT-3 levels in the ADHD group (4). These inconsistent findings in the literature suggest that the role of NT-3 in the pathophysiology of ADHD remains unclear. Although NT-3 is a neurotrophic factor that plays a critical role in processes such as neuronal differentiation, axonal guidance, and synaptogenesis, particularly during early neurodevelopmental periods, it has been suggested that its peripheral levels may remain relatively stable during later stages of development (48). Indeed, genetic studies have indicated a potential association between NT-3 and ADHD; however, this association does not appear to be consistently or directly reflected in clinical symptom severity or peripheral biomarker levels (24, 49, 50). In this context, the absence of a significant change in NT-3 levels in our study suggests that NT-3 may play a role in ADHD through mechanisms related to early developmental periods or genetic vulnerability rather than acute neurobiological processes. Moreover, the lack of significant associations between neurotrophic factor levels and clinical symptom severity assessed by Conners subscales supports the notion that these biological markers may reflect the neurodevelopmental and trait-level biological underpinnings of ADHD rather than momentary symptom burden. To more clearly elucidate the role of NT-3 in ADHD, further studies with longitudinal designs encompassing different age groups are warranted.

In our study, serum ACTH and cortisol levels were found to be significantly increased in the ADHD group compared with the healthy control group. This finding provides a novel and important contribution to the heterogeneous literature regarding HPA axis functioning in ADHD. Previous studies have demonstrated dysregulation of the HPA axis in children with ADHD, most commonly associated with abnormal or blunted diurnal cortisol rhythms (51). The elevated ACTH and cortisol levels observed in our ADHD sample may indicate a state of chronic stress capable of inducing alterations in brain functioning. Many studies in the literature have reported reduced morning cortisol levels or a flattened diurnal cortisol rhythm in ADHD, findings that have generally been interpreted as reflecting an insufficient stress response or a hypoactive HPA axis pattern. In contrast, some investigations have reported increased cortisol levels in specific ADHD subgroups, in the presence of concurrent environmental stressors, or during long-term assessments (29). The simultaneous elevation of ACTH and cortisol levels may suggest alterations in HPA-axis-related parameters in some individuals with ADHD; however, given the cross-sectional design and single morning measurements, no definitive conclusions can be drawn regarding HPA-axis hyperactivity. These findings imply that HPA axis functioning in ADHD cannot be adequately explained by a unidirectional hypoactivity model; rather, different HPA axis patterns may emerge depending on the clinical heterogeneity of the disorder, environmental stress load, and individual biological vulnerability. Given that studies directly assessing ACTH levels are relatively scarce in the literature, our findings also represent an original contribution in this regard (26, 30, 31). The increase in ACTH levels may be considered a peripheral reflection of stress-related CRH activation and may be associated with heightened biological sensitivity to environmental stressors in ADHD (52). Indeed, it has previously been suggested that psychosocial stress burden is increased in ADHD and that this may lead to chronic activation of physiological stress systems (9). Importantly, the observation of this finding in a medication-naïve sample allows the results to be interpreted independently of the potential secondary effects of psychostimulant treatment on the HPA axis, thereby strengthening the clinical and biological interpretability of our findings. Accordingly, our findings should be interpreted as evidence of possible alterations in HPA-axis-related parameters rather than definitive evidence of persistent HPA-axis hyperactivity.

The absence of a significant correlation between neurotrophic factors (BDNF, GDNF, VEGF, and NT-3) and HPA axis hormones (ACTH and cortisol) in this study may be considered a finding that partially contrasts with the biological interaction hypothesis proposed in the Introduction. However, this result does not indicate that these two biological systems operate entirely independently in ADHD; rather, it points to the bidirectional and complex nature of the interaction between the HPA axis and neurotrophins. Experimental and translational studies have shown that glucocorticoids can suppress the expression of neurotrophins, particularly BDNF, and that HPA axis hyperactivity may adversely affect neurotrophic support mechanisms (32, 41). Conversely, neurotrophins are also known to play an active role in the central regulation of the HPA axis. BDNF and other neurotrophins are highly expressed in brain regions that exert inhibitory control over the HPA axis, such as the hippocampus and prefrontal cortex, while they may be regulated in different directions in HPA axis–activating regions such as the amygdala. These region-specific regulatory mechanisms contribute to the adaptive or maladaptive shaping of the stress response and suggest that the neurotrophin–HPA axis interaction operates through a multilayered biological network rather than a unidirectional and linear relationship (53). In clinical samples, studies elucidating the direction and strength of this relationship are extremely limited. For instance, Bilgiç et al. reported strong negative correlations between BDNF, nerve growth factor (NGF), and NT-3 levels and ACTH and cortisol levels in medication-naïve children with obsessive–compulsive disorder (34). In contrast, the only study to date that has concurrently evaluated HPA axis hormones and neurotrophins in an ADHD sample did not examine correlations between these two biological systems (35). Unlike that study, we conducted correlation analyses in our sample; however, no significant associations were identified between neurotrophins and HPA axis hormones. This finding should not be interpreted as evidence that these two biological systems are entirely independent in ADHD. Rather, it suggests that their relationship may be too complex to be captured by simple correlations at the level of peripheral biomarkers and may be shaped by factors such as developmental stage, duration of stress exposure, and brain region–specific regulatory mechanisms (54). Within this framework, our findings support current views proposing that HPA axis hormones and neurotrophins may contribute to the pathophysiology of ADHD through parallel yet partially independent pathways. Future longitudinal and multilevel studies incorporating biochemical, epigenetic, and clinical measures are expected to provide a more detailed understanding of the dynamic interplay between these two biological systems.

The conduct of this study in a medication-naïve sample of children and adolescents, its status as one of the few studies to simultaneously and comprehensively evaluate neurotrophic factors (BDNF, GDNF, VEGF, and NT-3) and HPA axis hormones (ACTH and cortisol) in ADHD, and the exclusion of other psychiatric disorders through the administration of the K-SADS-PL constitute the main strengths of the study. Nevertheless, several limitations should also be acknowledged. First, the cross-sectional design of the study limits causal inferences and precludes conclusions regarding whether the observed biochemical alterations represent causes or consequences of ADHD. Second, HPA-axis functioning was assessed using a single morning measurement of ACTH and cortisol. This represents a significant methodological limitation because it does not capture the diurnal rhythm of cortisol, the cortisol awakening response, or broader temporal dynamics of HPA-axis regulation. Given the dynamic nature of the HPA axis, a single time-point measurement may not fully reflect the functional status of the system. Additionally, although age was controlled for in the analyses, pubertal stage was not systematically assessed. This should be considered a limitation, as pubertal development may affect neuroendocrine parameters, including HPA-axis hormone levels, and may contribute to between-subject variability. Third, although the sample size is comparable to that of many studies in the literature, it may have been insufficient to conduct detailed subgroup analyses according to ADHD subtypes. Finally, the absence of scales assessing anxiety and depressive symptoms should be considered another limitation, as it precludes the exclusion of potential effects of subclinical mood and anxiety symptoms.

## 5 Conclusion

This study provides a comprehensive biological profile in medication-naïve children and adolescents with ADHD by concurrently evaluating neurotrophic factors and HPA axis hormones. Our findings demonstrate that serum levels of BDNF, GDNF, VEGF, ACTH, and cortisol were significantly higher in the ADHD group compared with healthy controls, whereas NT-3 levels did not differ between groups. These results suggest that both neurotrophic mechanisms associated with neurodevelopmental processes and alterations in HPA-axis-related parameters, which are linked to stress-response regulation, may be involved in ADHD. However, the absence of significant correlations between neurotrophic factors and ACTH and cortisol levels indicates that these two biological systems may contribute to the pathophysiology of ADHD through parallel yet partially independent pathways. Overall, the findings support the notion that the biological underpinnings of ADHD are too complex to be explained by a single neuroendocrine or neurotrophic mechanism alone; rather, ADHD appears to be a neurodevelopmental disorder involving multiple, interacting systems. In this context, future longitudinal, subtype-specific, and multilevel studies incorporating repeated HPA axis measurements and biochemical, genetic, and clinical data are warranted to more clearly delineate the role of neurotrophins and stress response systems in ADHD.

## Data Availability

The raw data supporting the conclusions of this article will be made available by the authors, without undue reservation.

## Glossary

ADHD: Attention-deficit/hyperactivity disorder
HPA axis: Hypothalamic–pituitary–adrenal axis
BDNF: Brain-derived neurotrophic factor
GDNF: Glial cell line–derived neurotrophic factor
VEGF: Vascular endothelial growth factor
NT-3: Neurotrophin-3
ACTH: Adrenocorticotropic hormone
CRH: Corticotropin-releasing hormone
CPRS-SV: Conners’ Parent Rating Scale–Revised Short Version
K-SADS-PL: Schedule for Affective Disorders and Schizophrenia for School-Age Children – Present and Lifetime Version
BMI: Body mass index
ELISA: Enzyme-linked immunosorbent assay
ECLIA: Electrochemiluminescence immunoassay
MANCOVA: Multivariate analysis of covariance
ANCOVA: Analysis of covariance
ηp²: Partial eta squared
BBB: Blood–brain barrier
ASD: Autism spectrum disorder
